# Total hip replacement in adult patients with severe Kashin-Beck disease of the hip

**DOI:** 10.1186/s12891-016-1119-3

**Published:** 2016-07-15

**Authors:** Xin Tang, Jing Zhu, Zongke Zhou, Bin Shen, Pengde Kang, Fuxing Pei, Jian Li

**Affiliations:** Department of Orthopedic Surgery, West China Hospital of Sichuan University, No.37, Guoxue Alley, Chengdu, 610041 China; Respiratory and Thoracic Surgery Ward, West China Hospital of Sichuan University, No.37, Guoxue Alley, Chengdu, 610041 China

**Keywords:** Kashin-Beck disease, Replacement, Hip

## Abstract

**Background:**

The treatment of elderly patients with Kashin-Beck disease (KBD) remains clinically challenging, and clinical data are very lacking. The aim of this study was to retrospectively evaluate pain and functional outcomes following total hip replacement in adult patients with severe KBD of the hip.

**Methods:**

Twenty-two patients (32 hips) with KBD underwent primary hip replacement and were followed for at least 2 years. Radiographic and Clinical assessments were evaluated for each patient at 2 and 4 weeks and at 3, 6 and 12 months after the operation and annually thereafter. The efficacy index included the visual analogue scale (VAS) score, Harris hip score, functional score for adult Tibetans with Kashin-Beck Disease (FSAT-KBD) and radiographic outcomes.

**Results:**

The patients underwent a follow-up, and the mean follow-up time was 3.8 years. VAS scores significantly decreased within the first 6 months postoperatively. This decrease continued until the final follow-up (*p* < 0.01). This result was supported by a significant increase in the Harris and FSAT-KBD scores after the surgery (*p* < 0.01). At the final follow-up, there was no change in prosthesis positioning or radiographic evidence of prosthesis loosening. One case received impacted allograft bone croutons and had worn polyethylene components replaced after 6 years because the patient suffered severe pelvic and femoral osteolytic lesions postoperatively.

**Conclusions:**

Hip replacement can relieve pain and improve joint function in treating severe KBD hip. Additional studies that are more extensive are needed to confirm the findings of our study.

## Background

Kashin-Beck disease (KBD) is an endemic, chronic, osteoarticular disease that mainly occurs in China, Siberia and North Korea [1]. A recent study showed that there are more than 695,000 patients with KBD and that 105.84 million people are at risk for KBD in the endemic area of China [2]. KBD usually afflicts children between the ages of 3 and 12 years and becomes symptomatic in adults [3]. Although KBD has been studied for more than 100 years, little is known about its etiology. The current suspected causes mainly involve cereal contamination by mycotoxin-producing fungi, trace element nutritional deficiencies (selenium and iodine), high levels of fulvic acid in drinking water and harsh physical environments in the endemic areas [4–9].

The basic pathological feature of KBD is focal chondrocyte necrosis in multiple areas of hyaline cartilage, including articular cartilage and growth plates. The secondary pathological findings of KBD are the presence of repair and remodeling around the necrotic foci of the cartilage of the metaphysic and epiphysis, which can result in growth retardation, secondary degradation of cartilage and disability in the advanced stages of the disease, similar to that in osteoarthritis (OA) [10–12].

**Fig. 1:**
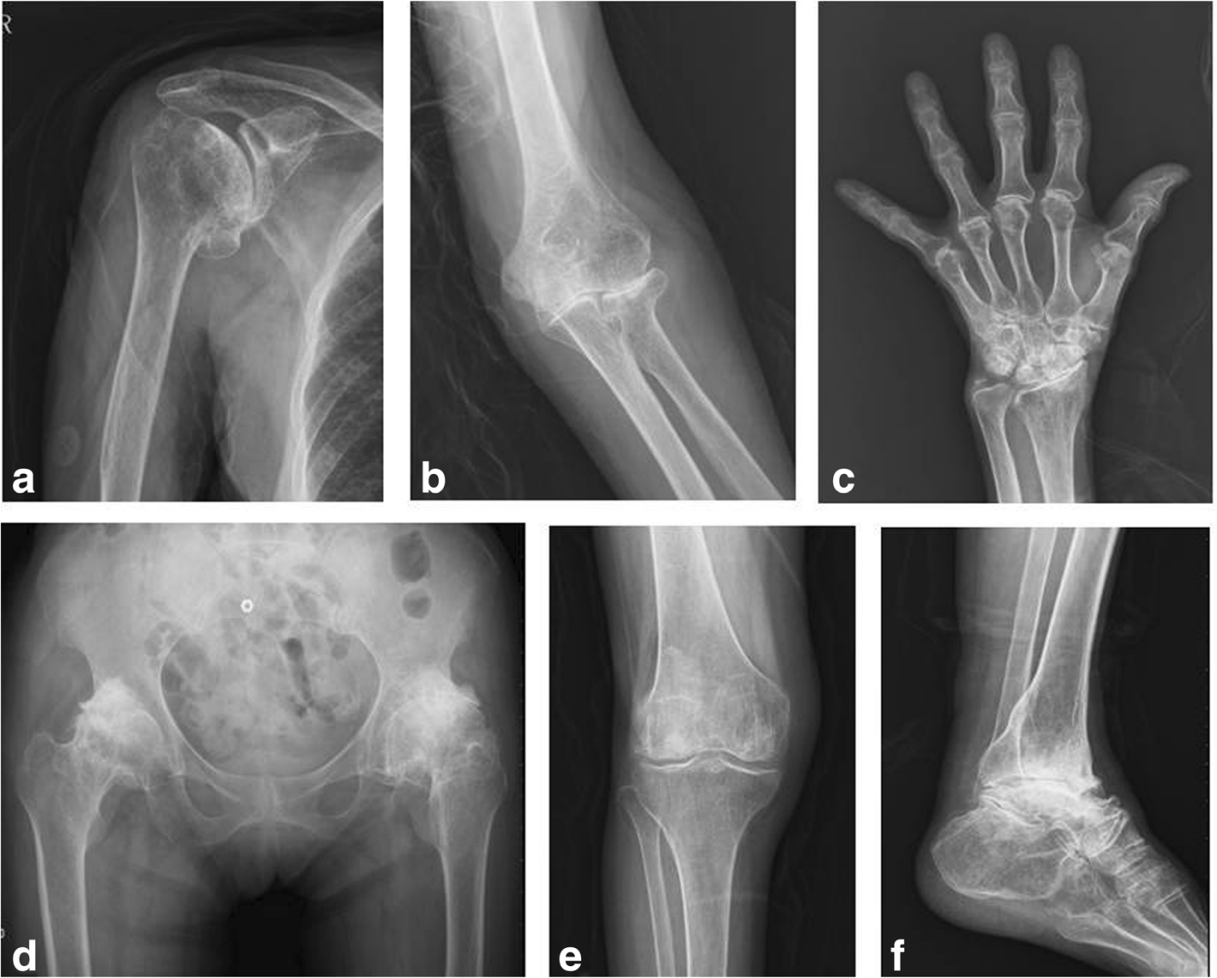
X-rays display the multiple joints affected in an elderly patient with end-stage KBD: **a** shoulder, **b** elbow, **c** wrist and fingers, **d** hip, **e** knee and **f** ankle

The clinical symptoms of KBD include pain in multiple joints, shortened and enlarged fingers and deformed joints involving limited activity in the end of the joints and leading to a high proportion of advanced-stage disability [13, 14] (Fig. 1). Pain and limited function of the hip are primary factors that affect the quality of life and functionality in elderly KBD patients [14].

Presently, no effective clinical measures can repair the cartilage damage or defects caused by KBD. Most treatment methods are derived from experience with OA treatment. The administration of non-steroidal anti-inflammatory drugs (NSAIDs), intra-articular injections of hyaluronic acid and physical therapy could reduce knee pain and/or improve knee joint function for elderly KBD patients prior to the advanced stage of KBD. However, these treatments do little to improve joint function and do not achieve long-term efficacy in end-stage KBD knee joints [15–18]. Moreover, only minimal data are available regarding improving the function of the hips in patients with severe KBD, especially when their hips have progressed to deformity.

Total hip replacement is a surgical procedure that has been shown to be very successful in relieving pain and physical functionality in older patients with advanced hip osteoarthritis (OA). In the United States, approximately 200,000 people receive total hip replacement every year [19], and that number is projected to increase to no less than 500,000 in 2030 [20, 21]. The Swedish Hip Arthroplasty Register reported that the 10-year survival rate of total hip replacement reached 96 % in both 2000 and 2002. In several studies, a 100 % survival rate has been achieved [22]. Few studies have reported clinical results in the treatment of late-stage KBD patients with total hip replacement. In this study, we retrospectively determined the effectiveness of total hip replacement for treating advanced hip disease (Kellgren-Lawrence (K-L) radiographic grade IV) in adult KBD patients and provided clinical data for the treatment of hip KBD in the future [23].

## Methods

This study was conducted at the Department of Orthopedics of West China Hospital of Sichuan University and was approved by the internal research administration department and ethics committee in accordance with the Helsinki Declaration. All the patients provided informed consent prior to enrollment in the study.

Patients were included if they had been symptomatic for no less than 6 months before enrollment, had experienced pain of more than 60 mm on a 100-mm visual analogue pain scale (VAS; 0 mm representing no pain and 100 mm representing the worst imaginable pain) in the target hip joint during assessment of the patient while walking, could not walk on a level floor for more than 200 m because of pain in the target hip, and had a K-L radiographic grade of more than III in the target hip [23]. Patients were excluded from the study for any unstable medical condition from a total hip replacement for the treatment of OA, such as inflammation, infection and abnormal vital signs.

At the time of screening, the medical history of each patient was recorded. A complete physical examination, particularly focused on the target hip and other affected joints, and laboratory assessments were performed. A radiograph of the target hip was also taken. Clinical diagnoses of KBD were made based on previously described clinical criteria by at least two senior orthopedic surgeons from the Orthopedics Department at the West China Hospital, West China Medical School, Sichuan University [11, 24].

Between 2007 and 2012, 22 patients who met the inclusion criteria underwent 32 primary total hip replacements in our department and were followed for more than 2 years. All operations were performed by the same group of surgeons. All of the patients were from Aba Prefecture, China, where a recent study reported that the mean incidence rate of KBD among adults is 41.0 % [25]. Table 1 presents the detailed data for these 22 cases in our study.

**Table 1:** KBD patients characteristics (*n* = 22, 32 hips)

Clinical manifestation	Mean ± SD/ratio
Age, yrs, mean ± SD	52.07 ± 17.46
Sex, M:F	15:7
BMI, kg/m2	23.85 ± 6.76
Side, R: L	18:14
Num of affected big joints	8.15 ± 4.25
KBD Stage, *n* (%)	
Stage I	9 (40.9 %)
Stage II	11 (50.0 %)
Stage III	2 (9.1 %)
Patient-assessed VAS score, mm	72.34 ± 9.16
Investigator-assessed VAS score, mm	71.41 ± 7.21
Harris (0–100)	44.88 ± 6.15
FSAT-KBD score (12–48)	27.69 ± 2.91

*KBD* Kashin-beck disease, *THA* total hip arthrosplast*y*, *BMI* body mass index, *R* right, *L l*eft, *VAS* visual analogue scale pain, *FSAT* functional score for adult Tibetans

### Surgical procedure

All total hip replacements were performed on the diseased hips by the same surgeons in laminar flow operating rooms. The patients received prophylactic antibiotics, namely, one dose (2.0 g) of intravenous cefazolin administered half an hour before the operation.

An appropriate implant was chosen via the preoperative use of templates, and a routine posterolateral approach was adopted. Vitalock cups were stabilized via a line-to-line reaming technique, and Duraloc cups were inserted via the press-fit technique.

All total hip replacements were performed using cementless implants. Small acetabular cups (cup size ≤ 46 mm) were placed in 13 hips after the deepening of the patient’s acetabulum. Six cups were reinforced using screws (4 hips with 2 screw and 2 hips with 3 screws). A morselized autogenous bone graft (resected from the femoral head) was used to fill cystic acetabular lesions and screw holes. Trochanteric osteotomies were used in the 3 hips in the high-dislocation group, and subtrochanteric osteotomies were used in the remaining 1 patient in the high-dislocation group.

Among the 22 patients, 10 patients with bilateral hip joint involvement underwent surgery for staged total hip replacements that were performed at an interval of approximately 7 days. Six patients with severe ipsilateral knee damage received total hip replacement first, followed by total knee replacement approximately 7 days later.

### Postoperative treatment

Postoperatively, one dose of prophylactic intravenous antibiotic (cefazolin, 2.0 g) was administered routinely. All the patients were administered an intramuscular injection of pethidine and/or oral NSAIDs to relieve post-surgery pain. All the patients received a nadroparin calcium injection to prevent venous thrombosis. Quadriceps, gluteal and hamstring setting exercises were started on the first postoperative day. The patients were encouraged to walk on the second postoperative day under the guidance of a physical therapist.

### Postoperative evaluations

All the patients received follow-up clinical and radiographic assessments at 2 and 4 weeks and at 3, 6 and 12 months after surgery and annually thereafter. During each follow-up, all the radiographic and clinical efficacy measures were evaluated at the outpatient clinics. The patients reported the clinical efficacy assessments, whereas the surgeon analyzed the radiographic results.

The outcomes of the clinical measures included complications during the perioperative period and VAS, Harris score [26], Functional Score for Adult Tibetans with Kashin-Beck Disease (FSAT-KBD) [27] and radiographic outcomes at every follow-up. The FSAT-KBD scale is developed by orthopedic experts in our department for the specific lifestyles of Tibetans. It was verified as a reliable, validated tool for the evaluation of improvements in KBD symptoms and function in Tibetans [28].

Postoperative radiographs were reviewed for evidence of prosthetic loosening according to the criteria of DeLee et al. and De Ranieri et al. [29, 30]. Cup and stem loosening or migration was measured between consecutive images using software on our hospital computer (Syngo Imaging V31, Siemens AG Medical Solutions, Germany). Normally, acetabular components were considered to be loosened if they had tilted by more than 5° in the abduction angle, migrated 2 mm or more or presented with a continuous radiolucent line that was more than 2 mm in width. The cementless femoral components were defined as being loosed if serial radiographs showed a progressive axial subsidence greater than 5 mm or a progressive change in the position of the femoral component. Osteolysis was defined as a circular or oval area of distinct bone loss and was graded as mild (< 1 cm^2^), moderate (1–1.9 cm^2^), or severe (> 2 cm^2^) on anteroposterior radiographs of the hips.

### Statistical Analysis

Statistical analyses were undertaken by SPSS 16.0 software (SPSS Inc., Chicago, IL, USA). Continuous data are presented as a mean ± standard deviation (SD). Changes in VAS, Harris and FSAT-KBD scores were analyzed using a general linear model and repeated-measures ANOVA. Multiple comparisons vs. baseline were assessed according to Dunnett’s T3 method. Comparisons of the acetabular component angles between the postoperative results and the final endpoints were analyzed using apaired-samples *t* test. A *p* value less than 0.05 was considered significant, and a *p* value less than 0.01 was considered highly significant for all statistical tests.

## Results

The mean follow-up time of the 22 patients (32 hips) who completed follow-up was 3.8 years (range: 2–7 years). All patients were followed for at least 2 years in the outpatient department after their operation. Seventeen patients (25 hips) were followed successfully in the outpatient department until their final follow-up. Five patients (7 hips) were followed via telephone, while the x-ray films were delivered, because of bad traffic conditions in the local area (a plateau mountain area with frequent storms in winter and spring).

**Fig. 2:**
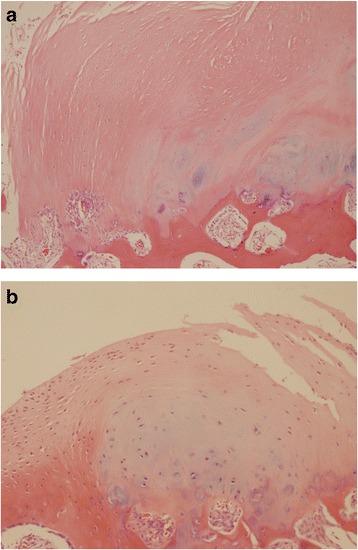
The histopathological findings display multiple focal areas of degeneration and surface fibrillation in the articular cartilage (**a**), endochondral ossification and loss of articular cartilage (**b**)

All of the patients were confirmed to have KBD based on pathological examinations after the operation. The main histopathological findings included multiple focal areas of degeneration and surface fibrillation in the articular cartilage, impaired chondrocyte differentiation (Fig. 2a), loss of articular cartilage, endochondral ossification and increased subchondral plate thickness (Fig. 2b).

### Perioperative complications

All the patients were required to walk postoperatively in an average of 2 days (range: 1–3 days). All the patients recovered uneventfully after surgery except for one 80-year old patient who developed a lung infection after the total hip replacement. This patient was treated with extended antibiotic therapy and encouraged to cough and breathe deeply. The lung infection was resolved within 1 week, and the patient successfully received a total knee replacement on the target knee. The patients received 5 days of postoperative care and were then discharged from the hospital in good health. No wound-healing disorders, wound infections or deep vein thromboses were recorded.

### Clinical efficacy measures

**Fig. 3:**
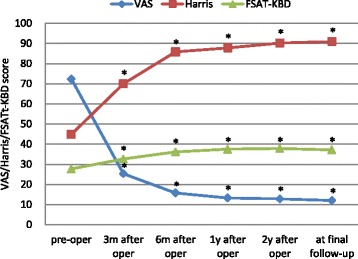
The VAS, Harris and FSAT-KBD scores for each time point. *p <0.01 compared with the pre-operation score

As measured by the VAS, Harris and FSAT-KBD scores, all the patients obtained a significant reduction in knee pain scores and a significant improvement in knee function after receiving total hip replacement (Fig. 3). The mean VAS score decreased from 72.34 ± 9.16 mm before surgery to 15.78 ± 4.23 mm at 6 months post-surgery (*p* < 0.01 vs. baseline), and this decrease was maintained through the final follow-up when the lowest score was reached (12.03 ± 2.80 mm, *p* < 0.01 vs. baseline). The average Harris score was significantly improved from 44.88 ± 6.15 before surgery to 85.81 ± 5.58 at 6 months after operation (*p* < 0.01 vs. baseline) and reached the highest score of 90.06 ± 4.57 at the final follow-up after total hip replacement (*p* < 0.01 vs. baseline). The mean FSAT-KBD score showed a trend similar to that of the Harris score and improved from 27.69 ± 2.91 at baseline to its peak of 36.81 ± 3.44 (*p* < 0.01 vs. baseline) at 2 years after the surgery. It decreased slightly to 36.66 ± 2.95 (*p* < 0.01 vs. baseline) at the final follow-up.

### Radiographic assessment

**Fig. 4:**
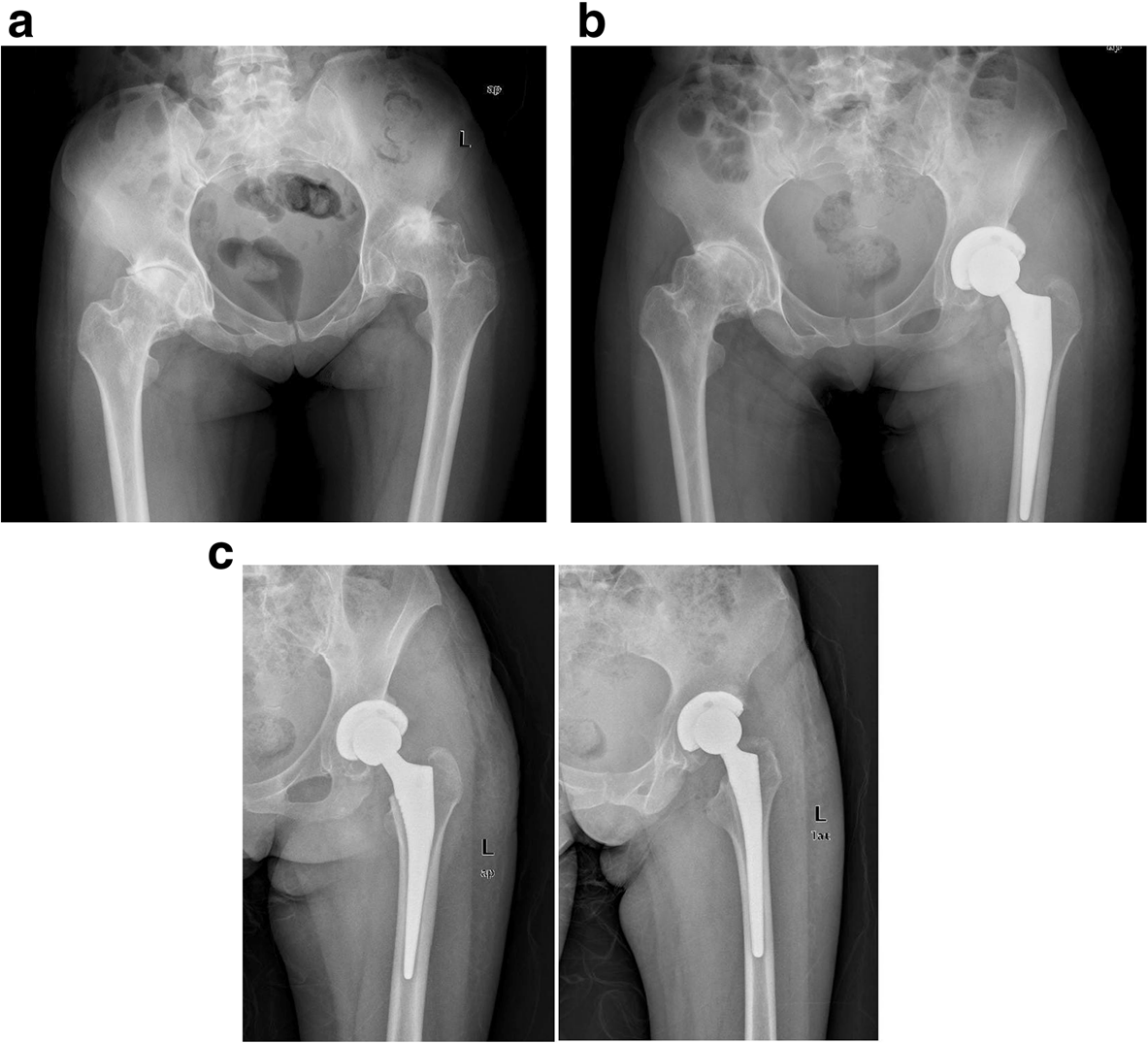
A male patient (bilateral hip with KBD) (**a**) with scores of 70, 43 and 25 points in the left hip on the VAS, Harris and Fast-KBD scales, respectively. He received total hip replacement in his left hip (**b**). Five-and-a-half years after the operation, there was no radiographic evidence of loosening or changes in component positioning (**c**), with scores of 10, 91 and 36 on the VAS, Harris and FSAT-KBD scales, respectively

The postoperative X-rays displayed that all the prosthesis components in the targeted hips were fixed well and were in a good position (Fig. 4).

At the first follow-up examination, the mean abduction angle of the acetabular component was 44.7° ±2.5° (range: 36.5–48.0°). At the last follow-up examination, the mean abductionangle of the acetabular components was 44.9° ± 2.4° (range: 37.1–48.3°). There was no significant difference in the abduction angle of the acetabular components (*P* =0.08). No gap was found at the interface between the bone and the acetabular components on the immediate postoperative radiograph (Fig. 4c). At the final follow-up, 2 sockets had radiolucent lines less than 2 mm in zone 1. Acetabular osteolysis was found in 12.5 % (4 hips) of the 32 hips (1 hip had severe osteolysis, 1 hip had moderate osteolysis, and 2 hips had mild osteolysis). The mean rate of polyethylene liner wear was 0.18 ± 0.14 mm/y (range: 0–0.24 mm/y).

After the operation, 25 femoral components were inserted in a neutral position, 4 in a mildly varus position, and 3 in a valgus position. At the latest follow-up examination, none of the femoral components were observed to have asignificant change in their position (Fig. 4c). Seven hips showed local osteolysis: in zone 1 in 2 hips and in zone 7 in 5 hips. The 3 hips with greater trochanteric osteotomies all acquired bony union.

One case experienced a severe hip osteolytic lesion (zone 1) complicated by femoral osteolysis (zone 7) 6 years postoperatively. The patient subsequently successfully received impacted allograft bone croutons in both the acetabular and femoral sides and had worn polyethylene components replaced in our department.

## Discussion

KBD is a disabling joint disease involving hyaline cartilage that typically leads to osteoarticular damage, deformity, pain and a severely limited range of activity in the advanced stages of the disease [31]. Based on our experience in treating hip patients with OA, we evaluated the effect of total hip replacement in treating serious KBD in the hips of adult patients. We found that total hip replacement could significantly relieve pain and improve the function of the target hip and overall quality of life. These results were supported by the significantly decreased VAS scores and the significantly increased Harris and FSAT-KBD scores 6 months after replacement, suggesting that total hip replacement provides long-lasting efficacy and treatment of the hip pain and activity limitations resulting from KBD.

Total hip replacement is a successful surgical procedure that could improve joint function and relieve pain involving the late stages of hip OA in adult patients. Good long-term survival has been reported for cemented and cementless total hip replacement implants in studies in America [32, 33]. Studies have shown that most patients achieved good results, with some showing a 100 % survival rate [22]. All the replacement procedures in our study were performed for the treatment of serious KBD hip with K-L grade IV. All the patients obtained good clinical results within 2 years postoperatively, and no change in the prosthesis positioning or radiographic evidence of prosthesis loosening was found at the last follow-up (range: 2–7 years), which is similar to the results of the aforementioned reports [22, 32, 33]. Only one case experienced a severe pelvic osteolytic lesion (zone 1) complicated with femoral osteolysis (zone 7) at 6 years postoperatively. This patient successfully received impacted allograft bone croutons and had worn polyethylene components replaced.

Few studies have reported clinical results on total hip replacement treatment in late-stage knee with KBD cases. Yang et al. reported nine cases of hip with KBD that included treatment with total hip replacement. Among these patients, 66.7 % of the patients (four cases) had good results, and one patient had fair (60–70 points) results based on the Harris scores [34]. More recently, Qian GB et al. reported on 13 cases (20 hips) of patients with KBD that underwent total hip replacement [35]. The 13 cases were divided into two groups based on the use of cemented or cementless components and were followed for 8–48 months. The outcomes showed that all the hips in both groups had good clinical outcomes according to their Harris scores. No radiographic evidence of prosthesis loosening, sinkage or change in prosthesis positioning at the end of the follow-up was reported. The final averaged Harris score was approximately 93.0 in these patients, which was similar to the final scores observed in our study.

KBD typically results in symmetrical and multi-joint damage in adult patients. Therefore, the function scales frequently used to evaluate global limb function might not be appropriate for evaluating the limb function of the KBD patients in our study because most items in these existing scoring systems, which have mostly been designed by Westerners, could be misunderstood and are confusing to Chinese KBD patients living in Aba Prefecture, Sichuan province, China. First, Aba Prefecture, where the patients worked, is a very poor mountainous region in China with harsh living conditions on the Tibetan plateau, known as the roof of the world, where life predominantly depends on agriculture and farming [9]. Second, most of the inhabitants in this epidemic area are Tibetan residents and follow Tibetan Buddhism, so they have a unique lifestyle and work habits that are significantly different from Western and urban residents. For example, the items in the scales designed by Westerners concerning whether one can use a knife and fork at the dinner table or whether one can sit on a chair may not be appropriate in this case because most KBD patients in this area use their right hand to manipulate food rather than using a knife and fork with both hands, and they primarily sit on the ground in a cross-legged position instead of sitting on chairs. The FSAT-KBD scale has been verified as a validated and reliable instrument to assess changes in KBD clinical symptoms and functional disability in Tibetans [28]. Our study showed the FSAT-KBD scores improved rapidly 3–6 months after hip replacement, suggesting that total hip replacement treatment could rapidly improve global joint function. The slight reduction in the FSAT-KBD scores after 2 years, relative to the 6-month scores, could be attributed to the worsening conditions of other limbs with the passage of the time [14, 27].

Some of the differences between KBD and OA are that OA usually occurs in elderly people with articular cartilage degeneration that is observable via radiology, it seldom affects multiple joints symmetrically and does not present with shortened fingers and phocomelic limbs. In contrast, KBD onset often occurs during childhood and adolescence, symmetrically affects multiple joints and mainly results in damage to the epiphysis, resulting in developmental disabilities that are observable via radiology [12]. Therefore, the overall functional results of KBD patients in the advanced stage are often worse than those of OA patients because systemic dysfunction may be aggravated by other large joints involved. KBD patients who undergo hip replacement often display the same trend of systemic functional results as time passes, which was confirmed in the present study by a slight decrease in the FSAT-KBD scores, relative to the 6 month scores, 2 years after undergoing total hip replacement.

The small number of patients included and the open-label design could be considered limitations of this study, which precludes any definitive conclusions regarding the effectiveness of total hip replacement treatment in KBD hip cases. We recommend additional randomized, long-term and prospective studies with more patients to confirm the findings in this study.

## Conclusions

Based on the results of our study, total hip replacement could be efficacious in treating serious KBD hips with promising clinical and radiographic outcomes at the intermediate follow-up period. Additional studies that are of higher quality are needed to confirm the findings in our study.

## Abbreviations

FSAT-KBD, Functional Score for Adult Tibetans with Kashin-Beck Disease, KBD, Kashin-Beck disease; K-L, Kellgren-Lawrence; NSAIDs, non-steroidal anti-inflammatory drugs; OA, osteoarthritis; SD, standard deviation; VAS, avisual analogue scale

## References

[1] Sokoloff L (1989). The history of Kashin–Beck disease. N Y State J Med.

[2] Zq Gao, X Guo, C Duan, W Ma, P Xu, W Wang, Jc Chen. Altered Aggrecan Synthesis and Collagen Expression Profiles in Chondrocytes from Patients with Kashin-Beck Disease and Osteoarthritis. Journal of International Medical Research 2012;40:1325. 10.1177/147323001204000411 22971484

[3] Hinsenkamp M (2001). Kashin-Beck disease. Int Orthop.

[4] Chasseur C, Suetens C, Michel V, Mathieu F, Begaux F, Nolard N (2001). A 4-year study of the mycological aspects of Kashine Beck disease in Tibet. Int Orthop.

[5] Haubruge E, Chasseur C, Debouck C, Begaux F, Suetens C, Mathieu F (2001). The prevalence of mycotoxins in KashineBeck disease. Int Orthop.

[6] Jirong Y, Huiyun P, Zhongzhe Y, Birong D, Weimin L, Ming Y (2012). Sodium selenium for treatment of KashineBeck disease in children: a systematic review of randomized controlled trials. Osteoarthritis Cartilage.

[7] Yao YF, Pei FX, Kang PD (2011). Selenium, iodine and the relation with Kashine Beck disease. Nutrition.

[8] La Grange M, Mathieu F, Begaux F, Durand MC (2001). KashineBeck disease and drinking water in Central Tibet. Int Orthop.

[9] Hinsenkamp M, Mathieu F, Claus W, Collard JF, de Maertelaere V (2009). Effects of physical environment on the evolution of KashineBeck disease in Tibet. Int Orthop.

[10] Malaisse F, Mathieu F (2008). Big bone disease. A multidisciplinary approach of KBD in Tibet Autonomous Region (P.R.China). LesPresses Agronomiques de Gembloux. ASBL (Belgium).

[11] Mathieu F, Begaux F, Lan ZY, Suetents C, Hinsenkamp M (1997). Clinical manifestations of Kashin–Beck disease in Nyemo Valley, Tibet. Int Orthop.

[12] Guo X, Ma WJ, Zhang F, Ren FL, Qu CJ, Lammi MJ (2014). Recent advances in the research of an endemic osteochondropathy in China: Kashin-Beck disease. Osteoarthritis Cartilage.

[13] Schepman K, Engelbert RH, Visser MM, Yu C, de Vos R (2011). Kashin Beck disease: more than just osteoarthrosis: a cross-sectional study regarding the influence of body function—structures and activities on level of participation. Int Orthop.

[14] Li Y, Zhou Z, Shen B, Yang J, Kang P, Yang X (2013). Clinical features of Kashin–Beck disease in adults younger than 50 years of age during a low incidence period: severe elbow and knee lesions. Clin Rheumatol.

[15] Bjordal JM, Ljunggren AE, Klovning A, Slørdal L (2004). Non-steroidal anti-inflammatory drugs, including cyclo-oxygenase-2 inhibitors, in osteoarthritic knee pain: meta-analysis of randomised placebo controlled trials. BMJ.

[16] Tang X, Pei FX, Zhou ZK, Liu G, Shen B (2012). A randomized, single-blind comparison of the efficacy and tolerability of hyaluronate acid and meloxicam in adult patients with Kashin-Beck disease of the knee. Clin Rheumatol.

[17] Yu FF, Xia CT, Fang H, Han J, Younus MI, Guo X (2014). Evaluation of the therapeutic effect of treatment with intra-articular hyaluronic acid in knees for Kashin-Beck disease: a meta-analysis. Osteoarthritis Cartilage.

[18] Mathieu F, Suetens C, Begaux F, De Maertelaer V, Hinsenkamp M (2001). Effects of physical therapy on patients with Kashin-Beck disease in Tibet. Int Orthop.

[19] Learmonth ID, Grobler GP, Dall DM, Jandera V (1995). Loss of bone stock with cementless hip arthroplasty. J Arthroplasty.

[20] Kurtz S, Ong K, Lau E, Mowat F, Halpern M (2007). Projections of primary and revision hip and knee arthroplasty in the United States from 2005 to 2030. J Bone Joint Surg Am.

[21] Lane NE (2007). Clinical practice. Osteoarthritis of the hip. N Engl J Med.

[22] Berli BJ, Schafer D, Morscher EW (2005). Ten-year survival of the MS-30 matt-surfaced cemented stem. J Bone Joint Surg Br.

[23] Kellgren JH, Lawrence JS (1957). Radiographic assessment of osteoarthritis. Ann Rheum Dis.

[24] Guo X (2001). Diagnostic, clinical and radiological characteristics of Kashin Beck disease in Shaanxi Province, PR China. Int Orthop.

[25] Li F, Deng J, Zhou D, Yang R, Li D (2005). Analysis on monitoring data of Kashin-Beck disease in Sichuan Province in 2005 (article in Chinese). J Prev Med Inf.

[26] Harris WH (1969). Traumatic arthritis of the hip after dislocation and acetabular fractures: treatment by mold arthroplasty. J Bone Joint Surg Am.

[27] Tang X, Zhou ZK, Shen B, Kang PD, Yang J, Li J (2014). Total knee arthroplasty in elderly patients with severe Kashin-Beck disease of the knee. Int Orthop.

[28] Huang Q, Zhou Z, Shen B, Yang J, Kang P (2010). The primary validation study of Kashin-Beck disease affected big joints function assessing system for adult Tibetans in Rangtang County (article in Chinese). Chin J Evid-based Med.

[29] DeLee JG, Charnley J (1976). Radiological demarcation of cemented sockets in total hip replacement. Clin Orthop Relat Res.

[30] De Ranieri A, Wagner N, Imrie SN, Hwang KL, Goodman SB (2011). Outcome of primary total hip arthroplasty in Charnley Class C patients with juvenile idiopathic arthritis: a case series. J Arthroplasty.

[31] Pasteels JL, Fu-De L, Hinsenkamp M, Rooze M, Mathieu F, Perlmutter N (2001). Histology of Kashin-Beck lesions. Int Orthop.

[32] Callaghan JJ, Bracha P, Liu SS, Piyaworakhun S, Goetz DD, Johnston RC (2009). Survivorship of a Charnley total hip arthroplasty: a concise follow-up, at a minimum of thirty-five years, of previous reports. J Bone Joint Surg Am.

[33] Della Valle CJ, Mesko NW, Quigley L, Rosenberg AG, Jacobs JJ, Galante JO (2009). Primary total hip arthroplasty with a porous-coated acetabular component: a concise follow-up, at a minimum of twenty years, of previous reports. J Bone Joint Surg Am.

[34] Yang L, Tu Z, Zhou J, Gao D, Dong S (2009). Surgical treatment on adult Kaschin-Beck disease (article in Chinese). Chin J Joint Surg (Electron Ed).

[35] Qian GB, Liu H, Zhang J, Wang P, Zhang YC, Zhang DP (2011). Comparison of different materials of total hip arthroplasty for reconstruction of hip function in adult Kashin-Beck disease (article in Chinese). J Clin Rehabil Tissue Eng Res.

